# Systematic Review of Exposure to Polycyclic Aromatic Hydrocarbons and Obstructive Lung Disease

**DOI:** 10.5696/2156-9614-11.31.210903

**Published:** 2021-08-17

**Authors:** Chinemerem C. Nwaozuzu, Kingsley C. Partick-Iwuanyanwu, Stephen O. Abah

**Affiliations:** 1 Africa Center of Excellence in Public Health and Toxicological Research, University of Port Harcourt, Port Harcourt, Nigeria; 2 Department of Biochemistry, University of Port Harcourt, Port Harcourt, Nigeria; 3 Department of Community Medicine, Ambrose Ali University, Ekpoma, Edo State, Nigeria

**Keywords:** polycyclic aromatic hydrocarbons, lung function, asthma, chronic obstructive pulmonary disease, COPD, FEV_1_ FVC, FEF_25–75%_

## Abstract

**Background.:**

There is fast-growing epidemiologic evidence of the effects of environmental chemicals on respiratory health. Polycyclic aromatic hydrocarbons (PAHs) have been linked with airway obstruction common in asthma and/or asthma exacerbation, and chronic bronchitis and emphysema.

**Objectives.:**

A systematic review of the association between exposure to PAHs and obstructive lung diseases is not yet available. The present systematic review aims to evaluate the evidence available in epidemiological studies that have associated PAHs with obstructive lung diseases such as asthma, chronic bronchitis, emphysema.

**Methods.:**

We performed a systematic literature search on PubMed, Google Scholar, and Scopus databases using relevant keywords and guided by predesigned eligibility criteria.

**Results.:**

From the total of 30 articles reviewed, 16 articles examined the link between PAHs and lung function in both adults and children. Twelve articles investigated the association between PAHs and asthma, asthma biomarkers, and/or asthma symptoms in children. Two articles studied the relationship between PAHs and fractional exhaled nitric oxide (FeNO), a biomarker of airway inflammation and the relationship between PAHs and obstructive lung diseases and infections, respectively. One study assessed exposure to daily ambient PAHs and cough occurrence.

**Discussion.:**

Twenty-seven studies found an association between PAHs and asthma and reduced lung function. In children it is reinforced by studies on prenatal and postnatal exposure, whereas in adults, reductions in lung function tests marked by low forced expiratory volume in 1 second, (FEV_1_), forced vital capacity (FVC), and forced expiratory flow (FEF_25–75%_) were the major health outcomes. Some studies recorded contrasting results: insignificant and/or no association between the two variables of interest. The studies reviewed had limitations ranging from small sample size, to the use of cross-sectional rather than longitudinal study design.

**Conclusions.:**

The literature reviewed in the present study largely suggest positive correlations between PAHs and obstructive lung diseases marked mainly by asthma and reduced respiratory function. This review was registered with PROSPERO (Registration no: CRD42020212894)

**Competing Interests.:**

The authors declare no competing financial interests.

## Introduction

Polycyclic aromatic hydrocarbons (PAHs) are a common group of environmental pollutants that usually occur as complex mixtures of over 300 organic compounds composed of multiple aromatic rings. Polycyclic aromatic hydrocarbons are compounds of interest due to their widespread presence as well as their carcinogenic, mutagenic and toxicologic potential.^1^

Globally, the negative impact of PAHs on health is a major public health concern. Apart from being linked to increasing risk of cancers,^2^ impaired neurodevelopment,^3^ genotoxicity,^4^ cardiovascular disease,^5^ metabolic syndromes,^6^ and recently onset of diabetes mellitus,^7^ PAH exposure may also lead to non-cancerous health effects, particularly respiratory diseases such asthma and chronic obstructive pulmonary disease (COPD).^8^–^10^

The World Health Organization (WHO) estimate indicates that more than 80% of deaths associated with chronic obstructive pulmonary disease (COPD) occurred in low- and middle income countries (LMIC) in 2019,^11^ although the burden of COPD attributable to PAHs remains unknown.

Human populations can encounter health risks from PAHs via a variety of exposure pathways, including inhalation of ambient air containing PAHs, e.g., tobacco smoke, contaminated air from incomplete burning of coal, consumption of charbroiled foods, and use of coal or wood stoves, fireplaces for cooking and residential heating, industrial processes, vehicle exhaust, fossil fuels, and dermal contacts with environmental media such as contaminated soil and water.^12^–^14^ The use of biomass (solid fuel) for cooking, lighting, and heating residence generates PAHs.^15^ Studies conducted by Pruneda-Alvarez *et al*. reported that women in developing countries using biomass as fuel in their home for cooking have greater exposure to PAHs than those who cook outside their homes or those who do not use biomass as fuel.^16^

Exposure to PAHs in adults seems to be linked to respiratory function and symptoms demonstrated as spirometric lung function parameters such as reduced forced expiratory volume in 1 second, (FEV_1_), which is the volume of air exhaled at the end of the first second of forced expiration; forced vital capacity (FVC), the volume of air that can be forcibly breathed out after taking the deepest breath possible and forced expiratory flow at 25 and 75% (FEF_25%–75%_) of the pulmonary volume.^17^–^19^ A comprehensive review by Lag *et al*. supports the notion that air-borne and particulate-bound PAH exposure may contribute to the development and/or exacerbation of respiratory disease and dysfunction.^20^ Jedrichowski *et al*. revealed a strong association between prenatal exposure to PAHs and particulate matter less than 2.5 micrometers in diameter (PM2.5) and asthma, wheezing, and cough in the first two years of life among children who were prenatally exposed.^21^ Huang *et al*. observed that an increase in concentration of urinary PAH metabolites was significantly associated with elevated risk of adult asthma.^22^

**Abbreviations**
*COPD*: Chronic obstructive pulmonary disease
*FEF*_*25–75%*_: Forced expiratory flow at 25–75%
*FEV*_*1*_: Forced expiratory volume in 1 second
*FVC*: Forced vital capacity
*OR*: Odds ratio

A cohort study by Zhou *et al*. reported negative associations between monohydroxy PAHs (OH-PAHs) and lung function. In this study, each unit increase in sum total of low molecular weight (LMW) and high molecular weight (HMW) – PAHs was associated with a decrease in FEV_1_ and FVC, respectively.^23^

Adverse respiratory health outcomes in children such as bronchitis have also been linked to PAH exposure.^24^–^25^ as well as decrements in lung function parameters.^26^–^29^ Reductions in the ratio of FEV_1_/FVC among adults in work settings was found to be linked with increased PAH exposures.^30^

Reviews exist on the association between PAHs in ambient air pollution and non-malignant respiratory diseases focusing on air pollution as the main source of exposure, but a comprehensive systematic review with a precise question about the link between obstructive lung diseases and PAHs exposure irrespective of the exposure route is non-existent. The purpose of this systematic review, therefore, was to evaluate the evidence available for an association between PAHs from all exposure routes (inhalation, ingestion, and dermal uptake) and obstructive lung diseases (asthma and COPD: chronic bronchitis and emphysema) as well as the strength of the association. The present study is expected to provide epidemiological evidence that could be useful in exploring the etiology of obstructive respiratory diseases.

## Methods

First, a search was performed on existing systematic reviews available in suitable electronic databases to ensure similar study had not yet been previously published to avoid duplication. A review protocol was then developed for our review question and the protocol was registered with PROSPERO (International prospective register of systematic reviews, 2020, CRD42020212894).^31^

### Study criteria and search strategy

The present study searched electronic databases of PubMed, Google Scholar, and Scopus in between August and September 2020. An advanced search builder was used to search in PubMed and Scopus. We considered studies with cohort, cross-sectional, case-control, and panel study designs, and articles written in the English language with no restrictions on publication date, and studies reporting both prenatal and post-natal exposure to PAHs, studies that examined exposure in children and adults with no age restrictions investigating the association between PAHs and obstructive lung diseases such as asthma and COPD. Studies were excluded if they were reviews, abstracts, editorials, commentaries, measured other pollutants aside from PAHs, or were published in languages other than English. The search strategy was modified when searching different databases. The following keywords were used to retrieve relevant articles:

“polycyclic aromatic hydrocarbons” OR “PAHs” OR “anthracene” OR “pyrene” OR “hydroxypyrene” OR “1-OHP” OR “benzo(a)pyrene” OR “BaP” OR “phenanthrene” OR “hydroxyphenanthrene” OR “fluorene” OR “hydroxyfluorene” OR “naphthalene” OR “hydroxynaphthalene” AND “obstructive lung disease” OR “asthma” OR “wheezing” OR “bronchitis” OR “emphysema” OR “COPD.”

In addition, the references of the retrieved articles were checked for additional studies. A reference manager (Endnote, version X9.2, Clarivate analytics, Philadelphia, USA) was used to manage the retrieved literature and to check for duplicates.

### Data screening and extraction

To select relevant articles, two members of the review team first reviewed the articles independently based on title and abstract. Having examined the titles, name of author, year of publication, journal name and issue number, duplicates were removed. We carefully screened articles again by titles and abstract, and finally by full texts available. Articles that were relevant to our objective were selected for inclusion while the others were excluded. Data from all the included studies were extracted with the help of a form predesigned by the reviewers *(Supplemental Material 1)* The data can be found in the summary of epidemiological studies included (*Supplemental Material 2*). Data includes: first author and publication year, study design, study participants and age at exposure, country of study or setting, exposure assessment, exposure metrics/study period outcome indicators, and key findings. The eligibility criteria mentioned earlier guided the entire screening process. Disagreements on article screening and data extraction were resolved by consulting with the third reviewer. Two reviewers disagreed on the inclusion of articles whose titles showed joint effects of PAHs and other pollutants. This was resolved by the third review who felt that those studies should be excluded on the grounds that the presence of other pollutants might have confounding effects on reported outcomes.

### Risk of bias assessment

Assessment of the quality of individual studies was done using the approach outlined by the Strengthening the Reporting of Observational Studies in Epidemiology (STROBE) checklist. The STROBE statement is a 22-item checklist used to effectively report observational studies. These items assessed the following sections of the articles: Title and abstract (item 1), Introduction (items 2 and 3), Methods (items 4–12), Results (items 13–17), Discussion (item 18–21), and other information (item 22 on funding).^32^

Using the 22-item checklist, two reviewers independently appraised the methodological quality of the included studies. For a study, each item was assigned “0,” “1” or a maximum score of “2,” depending on how they meet the requirements of each item. By assigning one score to each item, papers could get a total minimum score of 22 and a maximum score of 44. These ratings are provided in a Table (*Supplemental Material 3)*. Articles that scored 22 points and above were included in our review.

## Results

**Figure 1:**
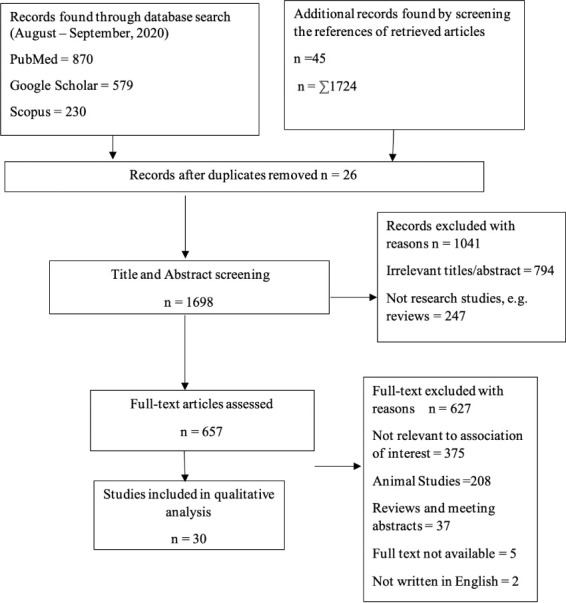
A Study Selection Flowchart

Study selection was done using an adapted PRISMA (Preferred Reporting Items for Systematic Reviews and Meta-analyses)^33^ diagram and shown in Figure 1. Studies included in our review are summarized in Supplemental Material 2, and the characteristics of included studies are summarized in Table 1.

**Table 1:** Characteristics of included epidemiological studies

**Study design**	**Study population**	**Setting**	**Exposure assessment**	**Outcome**	**Key findings**
Of all the study designs, cohort and cross-sectional studies were more frequent. There were 12 cross-sectional studies 17–18,26,29,34–41 11 cohort^19^,^21^,^27^–^28^,^30^,^42^–^47^ Four (4) case-controls^8^,^22^,^48^–^49^, and three (3) panel Studies^50^–^52^	The greatest number of participants is 15, 447 reported in a cross-sectional study by Liu *et al.,* 2015^39^ whereas the lowest number of participants was 64 reported by Barraza-Villarreal *et al.*^26^	Ten (10) studies were conducted in China^17^–^19^,^22^,^30^,^35^–^36^,^38^,^49^,^52^ followed by nine (9) studies^29^,^37^,^39^,^41^,^42^,^44^–^47^ performed in the United States while others were conducted in different regions of the world: Canada, India, Japan, Korea, Mexico, New Zealand, Poland, and Saudi Arabia	Eighteen (18) studies^18^–^19^,^22^,^26^–^27^,^30^,^34^–^37^,^39^–^41^,^46^,^49^, ^51^ detected PAHs using the urinary metabolites. Five (5) studies^17^,^29^,^42^,^50^,^52^ assessed PAHs by ambient (residential) air monitoring using air particle/PAHs sampler. One (1) study^38^ jointly used both urinary PAHs metabolites and ambient air monitoring. Five (5) studies^28^,^43^–^47^ measured both pre-natal and post-natal exposure by personal air monitoring of PAHs during the second or trimester of pregnancy and residential (indoor and outdoor) air monitoring during childhood up to 5–6 years of age respectively. Two studies^8^,^20^ by measuring serum PAHs levels and PAH-DNA adducts in umbilical cord blood respectively. Only one study measured blood PAHs level.	Twelve (12) studies^17^–^19^,^26^–^30^,^34^–^36^,^52^ reported health outcomes as lung functions assessed by their spirometric values. Eleven (11) studies^8^,^21^–^22^,^37^,^39^,^42^,^44^–^45^,^47^–^49^ reported health outcome as asthma. Two (2) studies^43^,^50^ reported chronic cough and number of wheezing days while two studies 38,50 reported increased exhaled nitric oxide, FeNO as the outcome. One (1) study^41^ reported asthma, chronic bronchitis and emphysema, wheezing, coughing, ear infection collectively. One (1) study^26^ also reported that decreased pH of EBC, a biomarker of airway inflammation as the health outcome.	Fourteen (14) studies 8,21,22,37,39,42–45,47–50 revealed positive associations between PAHs and diagnosed asthma, asthma biomarkers: IgE, IL-4, resistin anti-mouse IgE and asthma symptoms e.g chronic cough, wheezing, and shortness of breath. Thirteen (13) studies 17–19,26–30,34–37,52 reported inverse associations between PAHs and lung function parameters (FEV1, FVC, FEV1/FVC, FEF25–75%) as seen by reductions in spirometry test values performed. One (1) study^41^ showed a relationship between PAHs (2-hydroxyfluorene) and prevalent cases of chronic bronchitis and emphysema. Two (2) studies^38^,^50^ reported an association between PAHs and an increase in exhaled nitric oxide (NO) and (FeNO). One (1) study [26] found an association between PAH and PH of EBC, which is a biomarker of airway inflammation and increase in 2-hydroxyfluorene.

### Quality assessment of included studies

Supplemental Material 3 shows the quality assessment of studies included in this review. Of the 22 items in the STROBE checklist,^32^ all studies reported study designs, settings, and locations, exposure, diagnostic criteria, details of methods of assessment, and sources of data. All the cohort studies^19^,^21^,^27^–^28^,^30^,^42^–^47^ reported the sources of data, method of selection of participants, inclusion and exclusion criteria, method of follow-up, and follow-up period. Of the four case-control studies, only two^48^,^49^ gave eligibility criteria for case and control, sources of data and method of case ascertainment, and control selection. All twelve cross-sectional studies^17^–^18^,^26^,^29^,^34^–^41^ reported inclusion and exclusion criteria, source of data, and method of selection of participants. Seven studies^8^,^39^–^40^,^45^,^47^,^48^,^50^ did not explain how to control for confounding factors. It is not clear how studies dealt with readings of PAHs below the limit of detection except in Epton *et al*.^51^

## Discussion

Polycyclic aromatic hydrocarbons are widely distributed in the environment through various anthropogenic sources. Knowing the effects of PAHs on respiratory function is of particular interest due to their toxicological characteristics, especially carcinogenic, mutagenic, and teratogenic tendencies. Although PAHs are generally associated with obstructive respiratory diseases, there has been no systematic review assessing the strength of evidence behind this association. To the best of our knowledge, this is the first systematic review to evaluate the evidence of the association.

The conclusions from the array of included studies with regards to the association between exposure to PAHs and respiratory disease in humans were varied.

First, this present review found that many of the studies conducted at different times have the same positive relationships between PAHs and respiratory symptoms among different populations, using similar study designs, and that asthma was studied more than other obstructive lung diseases. The outcome of most studies showed greater odds of association between PAHs exposure and asthma as well as with development of asthma symptoms such as wheezing in individuals with and without preexisting asthma.^21^–^22^,^39^,^43^–^45^,^47^–^50^ For example, this is shown in the case-control studies by Huang *et al*.^22^ and Suresh *et al*.^48^

Huang *et al.* reported that PAHs: 2-hydroxyfluorene, 4-hydroxyphenanthrene, 1-hydroxyphenanthrene, and 1-hydroxypyrene were associated with elevated risks of asthma (odds ratio (OR) 2.04, 2.38, 2.04, and 2.35, respectively). Studies by Suresh *et al*. showed a high blood level of phenanthrene associated with bronchial asthma. (adjusted OR = 13.3, 95% confidence interval (Cl) 1.9–88.5) when compared with matched controls.

Another interesting finding is that prenatal exposure to PAHs can lead to respiratory symptoms during the early childhood in children exposed during pregnancy. Jedrichowski *et al.* reported that prenatal level of PAH-DNA (deoxyribonucleic) adducts correlated with wheezing days during the first two years of life (incident rate ratio: 1.69, 95% CI, 1.52 – 1.88).^21^ Polycyclic aromatic hydrocarbons also correlated with biomarkers of respiratory symptoms. Studies by Al-daghri *et al*., Li *et al*., and Anyenda *et al*. showed correlations with PAHs and immunoglobulin E IgE, interleukin 4 IL-4 and resistin, increased nitric oxide (NO) and fractional exhaled nitric oxide (FENO) which are biomarkers of childhood asthma and airway inflammation, respectively.^8^,^38^,^50^ However, Shiue *et al*. reported an inverse association between PAHs and asthma, although it showed a positive association between 2-hydroxyfluorene, 3-hydroxyfluorene and prevalent cases of emphysema. (OR: 1.60; 95% Cl: 1.26–2.03) and (OR: 1.42; CI, 1.15–1.77) respectively, and chronic bronchitis, (OR, 1.42, 95% CI, 1.04–1.94)

Secondly, the majority of the studies: (cross-sectional^17^–^18^, ^26^,^29^–^34^,^37^ and cohort studies^19^,^27^,^28^,^30^) found positive correlations between PAHs and reductions in lung function parameters: such as FEV_1_, FVC, and the ratio of both FEV/FVC, and FEF_25–75%_ after adjusting for possible confounders such as age, gender, dust exposure, body mass index (BMI) z-score, serum cotinine (a biomarker of passive tobacco smoke exposure), and family history of asthma. This finding is particularly important given that most of the studies in this review were related to asthma and thus provides evidence of possible association between PAH exposure and other obstructive airway diseases.

Third, the present study found that the claims of some studies in this review contradicted the general results. Rodriguez *et al.* reported no association between urinary 1-hydroxypyrene (1-OHP) concentration and respiratory function as lung functions were categorized as normal.^40^ Padula *et al.* reported that no association was observed with the sum total of 4,- 5,- and 6 ringed PAH, ΣPAH456 and respiratory functions among asthmatic children.^29^ Findings by Miller *et al* indicated that PAH metabolite concentrations were not associated with asthma or any of the respiratory symptoms examined.^46^ Rosa *et al.* claimed that prenatal PAH exposure alone was not associated with asthma nor IgE at 5–6 years among children not exposed to environmental tobacco smoke (OR, 0.65; 95% CI, 0.41–1.01).^47^ IgE is an immunoglobulin which facilitates type 1 hypersensitivity reactions and plays a significant role in the pathogenesis of allergic asthma. Serum IgE associates closely with the risk of asthma.^53^

Epton *et al.* recorded no significant difference in FEV_1_ between asthmatics and non-asthmatics although the ratio of FEV_1_/FVC was significantly lower in the asthmatic participants who were exposed to PAHs.^51^ Han *et al*. reported that although an increase in total PAHs was associated with reduced FEV_1_ in children with pre-existing asthma, there was no significant association between urinary PAHs and lung function among non-asthmatic children.^37^

The studies in this review have a number of strengths. A significant proportion of the studies (twenty-two studies)^17^–^19^,^21^,^22^,^27^,^29^,^30^,^34^–^37^,^39^,^41^,^42^–^47^,^49^,^52^ were conducted using a relatively large number of study participants ranging from 222 to 15 447. This has the statistical relevance of controlling for the risk of reporting false negative findings (type II error). It is well known that the generalizability of the results is limited by small sample size. In this review, small sample size (which ranged from 20 – 195) were found in eight studies.^8^,^26^,^28^,^38^,^40^,^48^,^50^,^51^

The power of a study is limited by small sample size which directly affects the statistical significance of some associations and the possible bias of study design. This can be said to be evident in studies by Li *et al*.^38^ and Rodriguez-Aguilar *et al*.^40^ Whereas Rodriguez *et al*. with a smaller sample size (134) found no association between 1-hydroxypyrene (1-OHP) and any respiratory symptoms, the study by Shen *et al.* with a larger sample size (505) found a strong association between 1-OHP and lower FEV_1_/FVC.^17^

Urinary assessment for PAHs is the most reoccurring means of exposure assessment in the studies reviewed. This is most likely due to its non-invasiveness and ease of collection which is convenient especially for studies involving a large number of participants. However, some studies^18^,^30^,^35^ opined that the use of single spot urine samples for exposure assessment has its limitations as it only reflects recent exposures but cannot indicate a past long-term level of PAH exposures. Repeated urine metabolite measurement and the use of 24-hour urine to measure past, chronic exposures were stated as preferable options to adequately describe this association.^18^,^30^ Theoretically, 24-hour void may be more reliable than spot urine, but it is not convenient to collect from study subjects and non-compliance can introduce bias in the sampling process. The use of first morning void as an alternative can be explained on the basis that it is often associated with 24-h void.^54^–^55^ Large epidemiological studies involving a large number of participants, may encounter difficulties trying to collect first-morning voids, hence, spot urine has been used as a more practical, less cumbersome method. Although PAHs are varied in nature, this review has observed that both the LMW-PAHs: naphthalene, fluorene, phenanthrene and HMW PAHs: those with 4 or more fused rings such as pyrene were associated with obstructive lung diseases.

Finally, we observed that majority of the studies were conducted in higher income countries, mainly China and the United States. It is therefore recommended that more studies be carried out in sub-Saharan African and other LMIC where there are often weak environmental pollution controls and more than 60% of heating and cooking fuel is derived from solid fuel such as coal and firewood.^15^

## Conclusions

Overall, the findings of the present review provide substantial evidence of the association between PAH exposure and obstructive lung diseases such as asthma, bronchitis, and emphysema, which is marked by reduced respiratory function. We therefore recommend that efforts be put in place to check future exposures, both prenatal/post and adult exposures, through policy making and other public health actions. Further research should focus on LMIC using longitudinal studies with long-term follow-up. This study can provide useful data in the evaluation of respiratory disease as patient exposures prior to disease onset is crucial to a fuller understanding of disease development.
