# Supplementary material for: Systematic Review of Exposure to Polycyclic Aromatic Hydrocarbons and Obstructive Lung Disease
Source: J Health Pollut. 2021 Aug 17;11(31):210903. doi: 10.5696/2156-9614-11.31.210903 (PMC8383797; doi:10.5696/2156-9614-11.31.210903)
Supplement: Supplementary file 1 [file Nwaozuzu_Supplemental_Material_1.docx]

**Supplemental Material 1**

**DATA EXTRACTION FORM**

This form was used to guide data extraction from studies for a systematic review on Exposure to Polycyclic Aromatic Hydrocarbons and Obstructive Lung Diseases.

| **Data to be extracted** | **Reviewer’s notes** | |
| --- | --- | --- |
| Title of study |  | |
| Author(s) |  | |
| Year of publication |  | |
| Type of participants/subjects |  | |
| Sample size |  | |
| Participant demographics |  | |
| Setting |  | |
| Time when study took place |  | |
| Exposure/association of interest |  | |
| Method of exposure assessment |  | |
| Method of lung function assessment |  | |
|  | | |
|  | Reason for Inclusion/exclusion | Reviewer’s decision |
| Type of study |  | Yes Include  No Exclude |
| Variables   1. Dependent: Obstructive lung diseases e.g., asthma, wheezing, asthma symptoms, emphysema, bronchitis, lower respiratory functions 2. Independent variables:   exposure to polycyclic aromatic hydrocarbons |  | Yes Include  No Exclude  ggggggggg |
| Reported key findings/outcome |  | Yes Include  No Exclude  ggggggggg |
| Comment(s) |  | |
