# Supplementary material for: Systematic Review of Exposure to Polycyclic Aromatic Hydrocarbons and Obstructive Lung Disease
Source: J Health Pollut. 2021 Aug 17;11(31):210903. doi: 10.5696/2156-9614-11.31.210903 (PMC8383797; doi:10.5696/2156-9614-11.31.210903)
Supplement: Supplementary file 2 [file Nwaozuzu_Supplemental_Material_2.docx]

**Supplemental Material 2**

**Summary of epidemiological studies included in the systematic review**

| **First author and publication year** | **Study design** | **Participants/age at**  **exposure** | **Setting** | **Exposure**  **assessment** | **Exposure metrics/study period** | **Outcome** | **Key findings** |  |
| --- | --- | --- | --- | --- | --- | --- | --- | --- |
| Anyenda (2013)**^50^** | Panel/  longitudinal study | 88 adult patients with chronic cough aged >20 years | Kanazawa, Japan | Daily ambient air monitoring of PAHs using air particle sampler | 6 months  (Jan. - June) | Chronic cough, exhaled NO | Ambient particulate PAH associated with occurrence of cough among adult chronic cough patients. Selected adjusted OR ratios for cough occurrence were 1.088 (95% CI, 1.031, 1.147); 1.209 (95% CI: 1.060, 1.379) per 1 ng/m^3^ increase for 2-day lag and 6-day moving average PAH exposure, respectively. Likewise, 5-ring PAHs had higher odds in comparison to 4-ring PAHs, P< 0.05.  Non-asthma group had slightly higher odds ratio for cough occurrence (OR, 1.127; 95% CI: 1.033, 1.228) per 1 ng/m^3^ PAH than asthmatics. |  |
| Al-Daghri (2013)**^8^** | Case-control | 195 Saudis children  98 asthma pediatric patients and 97 healthy controls | Saudi Arabia | Serum PAH | Not available | Asthma biomarkers | Strong associations between serum PAH levels and biomarkers of childhood asthma were detected. Serum PAHs (naphthalene, 4H-cyclobenta[def]phenanthrene, 1,2-benzanthracene, chrysene and benzo(e)  acephenanthrylene) had greater correlation with asthma biomarkers IgE, IL-4 and resistin in asthmatic children compared to non-asthma subjects (p = 0.004, 0.001 and 0.003, respectively). |  |
| Barraza-Villarreal (2014)**^26^** | Cross-sectional  (pilot study) | 64 school children aged 6-14 years | Mexico City, Mexico | Urine  PAH metabolite | Not available | Lung function (FEV_1_ and FVC)  Decreased pH of EBC (biomarker of airway inflammation) | Negative inverse association between increase in the concentration of 2-hydroxyfluorene and FEV_1_ (95% CL, -22.2 to -0.02), FVC. (95% CL, -22.9 to -0.2) and pH of EBC (95% CI: −0.066 to −0.005), *P* = 0.005. |  |
| Cakmak (2017)**^34^** | Cross-sectional | 3531 Canadian population aged 6-79 years | Canada | Urine PAH  Metabolite | Not clearly stated | Lung function FEV_1,_ FVC | Substantial reduction in FEV_1_ and FVC associated with an interquartile change in urinary PAH metabolites (95% CI -0.87, -0.25) for 4-hydroxyphenanthrene and (95% CI-1.28, -0.53) for 2-hydroxyphenanthrene. Significant reductions in FVC were associated with increases in the same eight PAHs, (95% CI -1.40, 0.68) for 2-hydroxyphenanthrene. |  |
| Cao (2020)**^35^** | Cross-sectional | 3367 Adult  residents, with mean age of 52 years | Wuhan, China | Urinary PAH metabolite | Not available | Lung function FEV_1,_ FVC | Increase in sum of urinary PAH metabolites was associated with a decrease in FEV_1_ and FVC (All P for trend < 0.05). | |
| Choi (2013)**^27^** | Cohort | 422 participants  ≥60 years | Seoul, Korea | Urinary PAH metabolite | 2008-2010 | Lung function  FEV_1_/FVC and FEF_25–75_ levels | Unit increase in urinary 1-hydroxypyrene levels was positively  associated with reduction in FEV1/FVC, and FEF_25–75_ levels  among the participants with at least one copy of TGA haplotype: namely: rs4646421 variants (CT+TT), rs4646422 wild-type (GG), and rs1048943 wild-type (AA),  (0.10–0.77%), 0.07–0.69%), and 0.08–0.80%) respectively.  No association was observed in those without the TGA haplotype | |
| Gale (2012)**^42^** | Prospective cohort | 315 asthmatic children from 6–11 years of age | California  United States | Air pollution monitoring in selected homes and central sites using PAH sampler | 2000-2008 | Asthma, increased wheeze | Association found between each of the three estimates of PAH exposure and increased wheeze. The odds ratios for  asthmatics exposed to PAHs (ng/m^3^) ranged from (OR, 1.01; 95% CI, 1.00-1.02) to (OR, 1.10; 95% CI, 1.04 - 1.17). | |
| Hou (2017)**^36^** | Cross-sectional | 2739 study participants aged 18-80 years | Wuhan, China | Urinary PAH metabolite | March-May, 2011 | Lung function marked by FEV_1_, FVC and the ratio of FEV_1_/FVC | A unit increment of log-transformed sum total of urinary metabolite (ΣOH-PAHs) concentration was related to a decrease in FEV1 in all participants. | |
| Han (2018)**^37^** | Cross-sectional study  2007-2012 | 2459 children aged 6-17 years | United Sates | Urinary  PAH | Not available | Asthma  % Predicted FEV_1_ | Children in the highest quartile of urinary Σmol3,4-PAH had 2.84 times higher odds of current asthma than those in the lowest quartiles of urinary Σmol3,4-PAH.  (95% [CI] for the odds ratio [OR] for 4th quartile vs 1st quartile = 1.20 - 6.70,  P = 0.02). Increase in sum of ⅀mol2-PAH associated with decrements in FEV_1_ among children with asthma. There was no significant association between urinary PAH and lung function among children without asthma. However, among children with asthma, each molar mass increase in ΣmolPAH was associated with 3.80% decrements in %  predicted FEV1 (95%CI = −6.68 to −0.88) and each molar mass increase in Σmol2-PAH were associated with 3.47% decreased %  predicted FEV1 (95%CI = −6.07 to −0.88). | |
| Huang (2018)**^22^** | Case-control | 503 cases and 536 matched controls | Wuhan, China | Urinary PAH | Not available | Asthma | A unit rise in log transformed 2-hydroxyfluorene (2-OHFLU), 4- hydroxyphenanthrene (4-OHPHE), 1-OHPHE, 2-OHPHE, 1-Hydroxypyrene (1-OHPYR) and sum total of PAH significantly associated with elevated risk of asthma with odds ratio of 2.04, 2.38, 2.04, 1.26, 2.35 and 1.34, respectively, P < 0.05. | |
| Jedrychowski, (2010)**^21^** | Prospective birth cohort | 339 children of 369 non-smoking mothers aged 18-35 years | Krakow, Poland | BaP-DNA adducts in cord blood | 2001-2004 | Asthma, early childhood wheeze | Prenatal level of PAH-DNA adducts correlated with wheezing days during the first two years of life, (incidence rate ratio (IRR) = 1.69, 95% CI, 1.52 – 1.88). | |
| Jedrychowski (2014)**^43^** | Prospective birth cohort | 257 newborns from non-smoking women aged 18-35 years. | Krakow, Poland | Personal monitoring of PAHs during pregnancy and post-natal indoor and outdoor residential air monitoring of PAHs | 2001-2004 | Number of wheezing days | Severity of wheezing days and recurrent wheezing reported in the follow-up associated positively with both prenatal and postnatal PAHs exposure. Odds ratio for severity of wheeze and prenatal PAH exposure was (OR, 1.53; 95%CI, 1.43 –1.64) and (OR, 1.13; 95%CI, 1.08 – 1.19) for postnatal PAH exposure. However, recurrent  wheezing was more strongly associated with airborne PAH levels measured at age 3 (OR, 2.31; 95%CI, 1.26 – 4.22) than transplacental PAH exposure (OR, 1.40, 95% CI; 0.85 – 2.09), but the  difference was statistically insignificant. | |
| Jedrychowski (2015)**^28^** | Prospective birth cohort  2001-2004 | 195 non-asthmatic children of non-smoking mothers | Krakow, Poland | Personal air monitoring in pregnancy and residential air monitoring for PAHs |  | Lung function by spirometry FEV_1_, FEF_25-75_,  FEV_05_ | Both prenatal and post-natal PAH exposure associated with reductions in FEV_05_, FEV_1,_ and FEF_25-75_  FEV05, FEV1 and FEF25–75 were inversely correlated with both prenatal and PAH levels (p = 0.013, p = 0.05, p = 0.013), respectively, and residential indoor PAH levels. | |
| Jung (2012)**^44^** | Prospective birth cohort | 349 children aged 5-6 years from 727 healthy, non-smoking Dominican or African American women | New York, USA | Personal air monitoring for prenatal exposure. and residential indoor monitoring for post-natal exposure up to 5-6 years | Not available | Asthma, wheeze | Recurrent high exposure to pyrene was associated with asthma [OR, 1.90; 95% CI, 1.13-3.20), medication use for asthma, wheeze, and emergency departmental visits. Among 242 nonatopic children, but not those sensitized to indoor allergens (n = 87) or with elevated total IgE levels (n = 171), high pyrene levels were associated positively with asthma (OR, 2.89; 95% CI, 1.77-5.69), asthma medication use (OR, 2.28; 95% CI, 1.13-4.59), and emergency department visits for asthma (OR, 2.43; 95% CI, 1.20-4.91). | |
| Li (2019)**^38^** | Cross-sectional  (Pilot study) | 20 postgraduate students with an average age of 24 years | Wuhan City, China | Urinary  PAH  outdoor and indoor PM2.5-bound PAHs monitoring | 4 seasons (Nov, Jan, March, June) | Increase in exhaled nitric oxide (FeNO) an indicator of airway inflammation | Doses of PM_2.5_-bound PAHs inhaled or urinary metabolites positively correlated with increased FeNO, an airway inflammation biomarker. Inhaled doses of PM2.5-bound PAHs or urinary OH-PAHs was positively related to increased FeNO, 13.5% (95% CI: 5.4- 22.2) at lag2 day or of 6.8% (95% CI: 3.4- 10.2) at lag1 day (All P < 0.05). | |
| Liu (2016)**^39^** | Cross-sectional study | 15 447 children:  7819 boys and  7628 girls aged 6-19 years | United States | Urinary  PAHs | NHANES 2001-2008 and NHANES 2011-2012 | Asthma, wheeze  ever cough | Remarkable association was found between urinary 2-phenanthrene and diagnosed asthma in boys (OR: 2.353, 95% CI: 1.156-4.792; P= 0.021) aged 13-19 years old. Positive association was observed between ever wheeze and 4-phenanthrene among girls aged 13-19 years (OR: 4.086, 95% CI: 1.326-12.584, P = 0.043). Moreover, an overall positive association between 1-pyrene and diagnosed asthma was observed. However, no association existed between levels of 1-napthol, 2-napthol, 3-fluorene, 2-fluorene, 3-phenanthrene, 1-phenanthrene or 9-fluorene with asthma or asthma symptom in this population. | |
| Miller (2004)**^45^** | Birth cohort | 303 non-smoking women residing in inner city with average age of 24.5±50 years | Northern Manhattan | Personal monitoring of airborne PAHs in the third trimester of pregnancy | Not available | Cough, difficulty breathing, wheeze, or probable asthma | Prenatal exposure to PAHs and early post-natal exposure to ETS associated with respiratory symptoms: difficultly breathing and probable asthma. By 12 months of age, more cough and wheeze were reported in children exposed to prenatal PAH in concert with ETS postnatally (PAH - ETS interaction odds ratios [ORs], 1.41 [p < 0.01] and 1.29 [p < 0.05], respectively). By 24 months, difficulty breathing and probable asthma were reported more frequently among children exposed to prenatal PAH and ETS postnatally (PAH _ ETS ORs, 1.54 and 1.64, respectively [p < 0.05]). | |
| Mu (2019)**^52^** | Panel study | 224 residents with mean age 5.34 | Wuhan, China | Personal PM_2.5_ monitoring using personal P.M_2.5_ samplers | Two study periods  2014–2015 and  2017-2018) | Respiratory functions  FVC and FEV_1_ | Increase of naphthalene, acenaphthene, fluoranthene and pyrene were associated with a decline in FVC and FEV_1._ Also, long-term high levels of three HMW-PAHs (benzo[a]anthracene, dibenzo[a,h]anthracene, and benzo[ghi]perylene) were associated with a decline in FVC. | |
| Padula (2015)**^29^** | Cross-sectional study | 467 asthmatic and  non-asthmatic children aged 9-18 years | Fresno, California, United States | Use of spatiotemporal model to measure daily 4-,5-,6-ringed PAHs | Not available | Reduction in lung function  FEV_1_ | Decrease in FEV_1_ for each 1 ng/m^3^ increase in sum total of 4-, 5-, and 6-ringed PAH among non-asthmatic children (95% CI: -0.20, -0.01) (p<0.01). However, no associations with the sum total of 4-, 5-, and 6-ringed PAHs (PAH456) were observed among asthmatic children. | |
| Rosa (2011)**^47^** | Prospective cohort | 290 children from 725 non-smoking healthy mothers | United States | Personal air monitoring of ⅀PAHs during pregnancy.  Prenatal + post-natal ETS defined by self-reported smokers. Plasma cotinine was measured in cord blood and child blood | Prenatal exposure was during the third trimester | Asthma,  wheeze,  total and specific serum IgE | Prenatal PAHs and prenatal ETS exposure were significantly associated with asthma at age 5-6 years (p < 0.05). However, prenatal PAH exposure alone was not associated with asthma nor IgE at the same age. (OR, 0.65; 95% CI, 0.41-1.01]). Among children exposed to prenatal ETS, a positive non-significant association was found between prenatal PAH exposure and asthma (OR, 1.96; 95% CI, 0.95-4.05). | |
| Shen (2018)**^17^** | Cross-sectional | 505  Male oven workers (390) 115 controls), age not available | China | Indoor and outdoor monitoring using an air particle sampler | Not available | Lung function  FEV_1_/FVC | Reduction of FEV_1_/FVC positively correlated with urinary 1-OHP in all subjects. (All *P <* 0.05). Inverse correlation of 1-OHP with FEV1/FVC in non-current smokers of exposure group was detected, (*P* = 0.043) | |
| Suresh (2009)**^48^** | Case -control | 42 cases of bronchial asthma and 20 controls | Lucknow, Northern India | Blood PAHs level | 2005 - 2006 | Bronchial asthma | High blood levels of phenanthrene associated with Bronchial asthma. (Adjusted OR = 13.3, 95% Cl: 1.9-88.5; P = 0.008) when compared with matched controls | |
| Shuie (2016)**^41^** | Cross-sectional | 5560 adults aged 20 -80 years | United States | Urinary PAH metabolites | 2011-2012 | Asthma, emphysema, chronic bronchitis,  wheezing, coughing,  ear infection | 2-hydroxyfluorene and 3-hydroxyfluorene positively associated with emphysema. (OR, 1.60, 95% CI 1.26-2.03, P = 0.001 and OR, 1.42, 95% CI, 1.15-1.77, P = 0.003, respectively) and chronic bronchitis, (OR, 1.42, 95%CI, 1.04-1.94, P=0.031 and OR, 1.40, 95%CI 1.03-1.91, P = 0.036, respectively). On the contrary, 1-hydroxyphenanthrene, 3- hydroxyphenanthrene, 1-hydroxypyrene, and 4- hydroxyphenanthrene) inversely associated with asthma and ear infections. No associations were found between wheezing, coughing, hay fever and PAHs. | |
| Wang (2016)**^30^** | Prospective cohort | 1243 coke oven workers | Wuhan, China | Urinary PAH | 2010-2014 | Lung function  FEV_1_/FVC and FEF_25%-75%_ | Faster decline in FEV_1_/FVC significantly associated with the sum total of PAHs (ΣOH-PAHs) Also, the baseline levels of urinary 1-OHNa, 1-OHPh, 2-OHPh, 9-OHPh, 1-hydroxypyrene (1-OHP), and ΣOH-PAHs were associated with significantly greater decline in FEF_25%-75%,_ (p < 0.05). | |
| Wang (2017)**^49^** | Case-control | 453 children  126 asthmatic children and 327 controls | China | Urinary PAH  metabolite | Not available | Asthma (cough, wheeze, and shortness of breath) | 1-OHP levels positively associated with asthma (OR, 1.42; 95% CI, 1.18–1.70) and increased serum IgE level, an asthma biomarker, (*p* = 0.05). | |
| Zhang (2017)**^18^** | Cross-sectional | 264 male workers, average age of 32  137 DEE-exposed workers and 127 controls | China | Urinary PAH metabolite | Not available | Lung function  FEV_1_/FVC  FEF75% | High urinary OH-PAH levels significantly correlated with a decrease of predicted % of FEV1, MMF, FEF_50%,_ FEF_75%_ in all study subjects. FEV1 and FEV_1_/FVC were significantly lower in the DEE-exposed workers compared with the controls, (p < 0.05) | |
| Zhou (2016)**^19^** | Cohort | 2747 participants  aged 18-80 years | Wuhan, China | Urinary PAH  metabolites | Not available | Lung function spirometry  FEV_1_ and FVC | Each 1-unit increase in the levels of total urinary metabolites and 2-OHNa, 2-OHFlu, 4-OHPh, 1-OHPh, 2-OHPh, and  ⅀PAH-OHs was associated with a reduction in FEV_1_ and FVC, (all P < 0.05). | |
| Epton (2008)**^51^** | Panel study | 93 male students aged 12 - 18 years | New Zealand | Urinary PAH |  | Asthma  ratio of FEV_1_/FVC in asthmatics | No significant difference in FEV_1_ between asthmatics and non-asthmatics, although FEV1/FVC ratio was significantly lower in the asthmatic students. | |
| Miller (2010)**^46^** | Prospective cohort | 222 children residing in inner city | New York, USA | Urinary PAHs metabolite | Not available | Asthma, wheeze, cough, bronchitis,  total and specific IgE | PAH metabolite concentrations not associated with asthma or any of the respiratory symptoms examined. However, increased 3- hydroxyfluorene and 3–hydroxyphenanthrene associated with higher anti-mouse IgE. | |
| Rodriguez-Aguilar (2019)**^40^** | Cross-sectional | 134 participants | San Luis Potosí, Mexico | Urine PAH metabolite | August -Nov. 2017 | Lung function parameter categorized as normal | No association between urinary 1-OHP concentration and respiratory function found, (1-OHP vs. FEV1 = Spearman Rho − 0.086, p = 0.419, 1-OHP vs. FEV1/FVC = Spearman Rho: − 0.081, p = 0.413) | |
