# Supplementary material for: Systematic Review of Exposure to Polycyclic Aromatic Hydrocarbons and Obstructive Lung Disease
Source: J Health Pollut. 2021 Aug 17;11(31):210903. doi: 10.5696/2156-9614-11.31.210903 (PMC8383797; doi:10.5696/2156-9614-11.31.210903)
Supplement: Supplementary file 3 [file Nwaozuzu_Supplemental_Material_3.docx]

**Supplemental Material 3**

**Bias risk of included studies**

|  |  | Anyenda *et al.* 2013 **^50^** | Al-Daghri *et al.* 2013 **^8^** | Barraza-Villarreal 2014 **^26^** | Cakmak *et al.* 2017 **^34^** | Cao *et al.* 2020 **^35^** | Choi *et al.* 2013 **^27^** | Gale *et al.* 2012 **^42^** | Hou *et al.* 2017 **^36^** | Han *et al.* 2018 **^37^** | Huang *et al.* 2018 **^22^** | Jedrichowski *et al.* 2010 **^21^** | Jedrychowski *et al.* 2014 **^43^** | Jedrychowski *et al.* 2015 **^28^** | Jung *et al.* 2012 **^44^** | Li *et al.* 2019 **^38^** | Liu *et al.* 2015 **^39^** | Miller *et al.* 2004 **^45^** | Mu *et al.* 2019  **^52^** | Padula *et al.* 2015 **^29^** | Rosa *et al.* 2011 **^47^** | Shen *et al.* 2018 **^17^** | Suresh *et al.* 2009 **^48^** | Shuie *et al.* 2015 **^41^** | Wang *et al.* 2016 **^30^** | Wang *et al.* 2017 **^49^** | Zhang *et al.* 2017 **^18^** | Zhou *et al.* 2016 **^19^** | Epton *et al.* 2008 **^51^** | Miller *et al.* 2010 **^46^** | Rodriquez-Aguilar 2019 **^40^** | % Score |
| --- | --- | --- | --- | --- | --- | --- | --- | --- | --- | --- | --- | --- | --- | --- | --- | --- | --- | --- | --- | --- | --- | --- | --- | --- | --- | --- | --- | --- | --- | --- | --- | --- |
| 1 | Title & abstract | 2 | 2 | 2 | 1 | 1 | 1 | 2 | 1 | 2 | 2 | 2 | 2 | 2 | 1 | 2 | 1 | 2 | 2 | 2 | 1 | 1 | 2 | 1 | 2 | 2 | 2 | 1 | 2 | 2 | 1 |  |
| 2 | Background/rationale | 2 | 2 | 2 | 2 | 2 | 2 | 2 | 2 | 2 | 2 | 2 | 2 | 2 | 2 | 2 | 2 | 2 | 2 | 2 | 2 | 2 | 2 | 2 | 2 | 2 | 2 | 2 | 2 | 2 | 2 |  |
| 3 | Objectives | 2 | 2 | 2 | 1 | 1 | 2 | 2 | 0 | 2 | 1 | 2 | 1 | 2 | 2 | 1 | 1 | 2 | 1 | 0 | 2 | 1 | 2 | 1 | 1 | 2 | 1 | 1 | 1 | 2 | 2 |  |
| 4 | Study design | 2 | 2 | 2 | 2 | 2 | 2 | 2 | 0 | 2 | 2 | 2 | 2 | 2 | 2 | 2 | 2 | 2 | 2 | 2 | 2 | 2 | 2 | 2 | 2 | 1 | 2 | 2 | 2 | 2 | 0 |  |
| 5 | Setting | 1 | 1 | 1 | 2 | 1 | 2 | 2 | 2 | 2 | 2 | 2 | 2 | 2 | 2 | 2 | 2 | 2 | 2 | 2 | 1 | 1 | 2 | 2 | 2 | 1 | 1 | 2 | 1 | 2 | 2 |  |
| 6 | Participant | 1 | 1 | 2 | 2 | 2 | 2 | 2 | 2 | 2 | 2 | 2 | 1 | 1 | 2 | 2 | 1 | 2 | 2 | 1 | 1 | 2 | 2 | 1 | 2 | 2 | 2 | 2 | 2 | 2 | 2 |  |
| 7 | Variables | 1 | 2 | 2 | 2 | 2 | 2 | 2 | 2 | 2 | 2 | 2 | 2 | 2 | 2 | 2 | 2 | 1 | 2 | 2 | 2 | 2 | 2 | 2 | 2 | 1 | 2 | 2 | 2 | 2 | 2 |  |
| 8 | Data source/measurement | 2 | 2 | 2 | 2 | 2 | 2 | 2 | 2 | 2 | 2 | 2 | 2 | 2 | 2 | 2 | 2 | 2 | 2 | 2 | 2 | 2 | 2 | 1 | 2 | 2 | 2 | 2 | 2 | 2 | 2 |  |
| 9 | Bias | 0 | 0 | 2 | 2 | 2 | 2 | 2 | 1 | 2 | 2 | 2 | 2 | 1 | 2 | 2 | 1 | 1 | 1 | 2 | 1 | 2 | 0 | 2 | 2 | 2 | 2 | 2 | 2 | 2 | 0 |  |
| 10 | Study size | 0 | 0 | 0 | 2 | 2 | 2 | 2 | 2 | 2 | 2 | 2 | 0 | 0 | 2 | 1 | 1 | 2 | 2 | 2 | 1 | 2 | 0 | 1 | 1 | 1 | 1 | 2 | 0 | 2 | 2 |  |
| 11 | Quantitative variable | 1 | 2 | 2 | 2 | 2 | 2 | 2 | 2 | 2 | 2 | 2 | 1 | 2 | 2 | 1 | 2 | 2 | 2 | 2 | 1 | 2 | 1 | 2 | 2 | 2 | 2 | 2 | 2 | 2 | 1 |  |
| 12 | Statistical methods | 1 | 1 | 1 | 1 | 2 | 2 | 2 | 2 | 2 | 2 | 2 | 1 | 1 | 2 | 1 | 1 | 1 | 1 | 2 | 1 | 1 | 0 | 1 | 1 | 2 | 2 | 2 | 1 | 1 | 0 |  |
| 13 | Participants | 1 | 0 | 0 | 1 | 1 | 2 | 1 | 2 | 2 | 1 | 0 | 1 | 1 | 2 | 1 | 0 | 2 | 1 | 1 | 0 | 1 | 2 | 0 | 1 | 1 | 0 | 1 | 0 | 1 | 2 |  |
| 14 | Descriptive data | 2 | 1 | 1 | 1 | 2 | 2 | 1 | 2 | 2 | 2 | 2 | 1 | 1 | 2 | 1 | 1 | 2 | 2 | 1 | 1 | 2 | 2 | 2 | 1 | 1 | 2 | 2 | 1 | 1 | 2 |  |
| 15 | Outcome data | 1 | 1 | 2 | 2 | 2 | 2 | 2 | 2 | 2 | 2 | 2 | 2 | 2 | 2 | 1 | 2 | 2 | 2 | 2 | 2 | 2 | 0 | 2 | 2 | 2 | 2 | 2 | 1 | 2 | 0 |  |
| 16 | Main results | 2 | 0 | 2 | 2 | 1 | 2 | 2 | 2 | 2 | 1 | 2 | 1 | 2 | 2 | 1 | 2 | 1 | 0 | 2 | 2 | 1 | 0 | 2 | 2 | 2 | 1 | 2 | 0 | 2 | 0 |  |
| 17 | Other analyses | 2 | 1 | 1 | 2 | 2 | 2 | 2 | 2 | 2 | 1 | 2 | 1 | 1 | 2 | 0 | 1 | 2 | 2 | 2 | 1 | 1 | 2 | 2 | 2 | 2 | 2 | 2 | 1 | 1 | 2 |  |
| 18 | Key results | 2 | 2 | 2 | 2 | 2 | 2 | 1 | 2 | 2 | 2 | 2 | 2 | 2 | 2 | 2 | 2 | 2 | 2 | 2 | 2 | 2 | 2 | 2 | 2 | 1 | 2 | 2 | 2 | 2 | 2 |  |
| 19 | Limitations | 2 | 0 | 2 | 2 | 2 | 2 | 2 | 2 | 2 | 2 | 2 | 2 | 0 | 2 | 2 | 2 | 2 | 2 | 2 | 2 | 2 | 2 | 2 | 2 | 2 | 2 | 2 | 2 | 2 | 2 |  |
| 20 | Interpretation | 2 | 1 | 2 | 2 | 2 | 2 | 2 | 2 | 2 | 2 | 2 | 2 | 2 | 2 | 2 | 2 | 2 | 1 | 2 | 2 | 2 | 2 | 2 | 2 | 1 | 2 | 2 | 2 | 2 | 2 |  |
| 21 | Generalizability | 2 | 2 | 2 | 2 | 2 | 2 | 2 | 2 | 2 | 2 | 2 | 2 | 2 | 1 | 1 | 2 | 2 | 1 | 1 | 0 | 2 | 2 | 2 | 2 | 2 | 2 | 2 | 2 | 0 | 2 |  |
| 22 | Funding | 2 | 0 | 0 | 0 | 2 | 0 | 0 | 2 | 0 | 0 | 0 | 0 | 0 | 2 | 2 | 0 | 0 | 2 | 0 | 0 | 0 | 0 | 0 | 2 | 0 | 0 | 0 | 0 | 2 | 0 |  |
|  | **Score** | **33** | **25** | **34** | **36** | **38** | **41** | **39** | **38** | **42** | **38** | **40** | **32** | **32** | **42** | **33** | **32** | **38** | **37** | **36** | **29** | **35** | **31** | **34** | **39** | **34** | **36** | **39** | **30** | **38** | **30** |  |
